# Prognostic significance of carotid and vertebral ultrasound in ischemic stroke patients

**DOI:** 10.1002/brb3.475

**Published:** 2016-04-27

**Authors:** Antonio Muscari, Andrea Bonfiglioli, Donatella Magalotti, Giovanni M. Puddu, Veronica Zorzi, Marco Zoli

**Affiliations:** ^1^ Stroke Unit – Medical Department of Continuity of Care and DisabilityS.Orsola‐Malpighi HospitalUniversity of BolognaBolognaItaly; ^2^ Department of Medical and Surgical SciencesS.Orsola‐Malpighi HospitalUniversity of BolognaBolognaItaly

**Keywords:** Carotid and vertebral ultrasound, dependency, follow‐up studies, ischemic stroke, modified Rankin scale, mortality, outcome, prognosis

## Abstract

**Objectives:**

The ultrasound investigation of carotid and vertebral arteries is routinely performed in stroke patients to determine the etiopathogenetic classification and possible need of revascularization. However, the medium and long‐term prognostic implications of carotid and vertebral ultrasound in ischemic stroke patients are not yet known.

**Methods:**

This study included 309 ischemic stroke patients (mean age 76.3; 160 men). They all had undergone carotid and vertebral ultrasound (carotid stenoses were measured according to the European Carotid Surgery Trial [ECST] method). After a median interval of 9.4 months, a telephone follow‐up was performed to determine their outcome. Dependency or death (modified Rankin scale‐mRS >2) and all cause mortality were the study end‐points.

**Results:**

At follow‐up, 158 patients had a mRS >2. In multivariate analysis, of 13 variables univariately predictive of dependency or death, only National Institutes of Health Stroke Scale (NIHSS) score (*P* < 0.0001), age (*P* < 0.0001) and ipsi‐ or contralateral carotid stenosis ≥60% (O.R. 3.5, 95% C.I. 1.5–8.6, *P* = 0.006) remained associated with a mRS >2. Sixty‐nine patients had died. In a Cox proportional hazards regression, of 10 variables univariately predictive of mortality, only NIHSS score (*P* < 0.0001), age (*P* = 0.003), total anterior circulation syndrome (*P* = 0.004), vertebral Doppler abnormalities (O.R. 2.2, 95% C.I. 1.3–3.6, *P* = 0.006), male sex (*P* = 0.02), and hypercholesterolemia (*P* = 0.04, inverse relationship) remained associated with mortality.

**Conclusions:**

In stroke patients, carotid stenoses ≥60%, ipsi‐ or contralateral to cerebral lesions, were associated with an increased medium and long‐term probability of dependency or death, and abnormalities of vertebrobasilar flow were a significant indicator of death risk, independent of stroke severity and age.

## Introduction

The ultrasound investigation of carotid and vertebral arteries is now routinely performed in all ischemic stroke patients. Its main functions are the identification of significant carotid stenoses (which sometimes require urgent revascularization procedures) and the etiologic classification of stroke according to TOAST criteria (Adams et al. 1993), the stroke may be attributed to a carotid atherosclerotic lesion when the latter causes a stenosis ≥50% and is ipsilateral to the cerebral lesion). The flow abnormalities in the vertebrobasilar system have fewer practical implications; nevertheless they may suggest further investigations to study the posterior circulation. Even in the absence of abnormalities, carotid and vertebral ultrasound can provide useful diagnostic elements, favoring stroke classification within different pathogenetic categories (small artery, cardioembolism, or cryptogenic stroke).

Despite the evident short‐term prognostic significance of demonstrating a relevant carotid lesion (Rothwell 2008), so far no study has examined the medium and long‐term prognostic significance of carotid and vertebral ultrasound abnormalities in ischemic stroke patients.

Thus, we have re‐examined the reports of the ultrasound investigations performed in our Stroke Unit during nearly 2 years of activity, and have related them to the information obtained by a telephone follow‐up interview performed on average 9 months after hospitalization. This study showed that some parameters of carotid and vertebral ultrasound are predictive of medium and long‐term disability and mortality in ischemic stroke patients.

## Methods

### Patients

We examined the medical records of the ischemic stroke patients admitted to the Stroke Unit of the S.Orsola‐Malpighi Hospital in Bologna, Italy, from February 10, 2007 to December 9, 2008 (*N* = 371). Thirty of these patients had not undergone a carotid and vertebral ultrasound. The remaining 341 patients were called by telephone on average 9.4 months after the ultrasound investigation, but 32 of them were not found (they never answered the phone or their phone number had been disconnected). Thus, the study eventually included 309 patients – mean age 76.3 ± 11.2 years, 160 men (52%) – who had undergone a carotid and vertebral ultrasound during their stay in stroke unit and who could be contacted for a follow‐up telephone interview.

Both the mean age (73.8 ± 14.4 years) and the sex distribution (27 men, 44%) of the 62 patients that were not included in the final sample did not differ from those of the 309 participants (respectively, *P* = 0.19 and *P* = 0.24). Non participants had an apparently (but not significantly) more severe stroke. In particular, the total anterior circulation syndromes (TACS) were 21/62 (34%) among the non‐included patients and 72/309 (23%) among the included patients (*P* = 0.08). The deaths in stroke unit were 4/62 (10%) versus 10/309 (3%), *P* = 0.23.

Ischemic stroke was defined according to the sudden appearance of neurological symptoms lasting more than 24 h, in the absence of hemorrhagic signs on the first brain CT scan.

The second CT scan, performed on average 3 days after admission, allowed the identification of the ischemic lesion and the measurement of its maximum diameter on CT slices.

The follow‐up telephone interview was done with the patient or a proxy. As previously reported (Muscari et al. 2011), the operator was not aware of the baseline variables and used a flow‐chart of five standardized questions that univocally and reproducibly led to the modified Rankin scale (mRS) score (Rankin 1957): (1) Can you walk without the help of another person ? If NO → (2) Are you bedridden needing help for everything? If YES → mRS = 5, if NO → mRS = 4. If the answer to question 1 was YES → (3) Do you need the help of other persons for some daily activity? If YES → mRS = 3, if NO → (4) Can you do everything you were able to do before the stroke? If NO → mRS = 2, if YES → (5) Are you completely restored as before the stroke? If YES → mRS = 0, if NO → mRS = 1.

In case of death of the patient, the date of death was requested (this was not provided in three cases).

Because of the retrospective nature of this study, a written consent could not be obtained from the patients. However, the analysis of the data collected in our data base for study purposes was authorized by our Hospital Direction, and a verbal consent was obtained from the patients or their proxies at the moment of the telephone follow‐up.

### Clinical variables

All patients underwent a standardized neurological examination on admission to the Stroke Unit, and a score according to the National Institutes of Health Stroke Scale (NIHSS) was obtained which allowed the quantification of stroke severity (Lyden et al. 1999).

The patients under antihypertensive treatment, or with average blood pressure values ≥140/90 mmHg during the first 48 h of stay, were considered hypertensive.

The patients under treatment with a statin, or with total cholesterol level ≥200 mg/dL, were considered hypercholesterolemic.

The patients under antidiabetic treatment, or with fasting blood glucose level ≥126 mg/dL, were considered diabetic.

The patients who had smoked at least 100 cigarettes during their life and who had not smoked during the last month before the stroke were considered ex‐smokers, while the patients who had smoked at least 100 cigarettes in their life and had smoked during the last month before the stroke were considered current smokers. Ex‐smokers plus current smokers formed the group of ever smokers.

As far as the etiopathogenetic mechanism of stroke is concerned, patients were classified into five categories according to the Trial of ORG 10172 in Acute Stroke Treatment (TOAST) definitions (Adams et al. 1993): large artery lesion, cardioembolism, small (perforating) artery impairment, other determined cause, and undetermined cause.

### Carotid and vertebral ultrasound assessment

All patients underwent a carotid and vertebral ultrasound assessment, which was performed by a conventional ultrasound system (Esaote‐MyLab 75, Esaote SpA, Genoa, Italy) equipped with a multi‐frequency (7.5–13 MHz) linear array transducer.

Two experienced ultrasound operators (A.B. and D.M.) performed all the investigations and reviewed the stored ultrasound images blinded to follow‐up results.

Carotid plaques were defined according to the presence of an intimal thickening ≥2 mm. Plaque structure was assessed in B‐mode and defined according to its echogenicity: hypoechogenic (soft), hyperechogenic (dense), calcific, or mixed/nonhomogeneous. The severity of carotid stenosis was graded according to the ECST method (Wardlaw and Lewis 2005). In brief, the carotids were insonated throughout their length and, from a longitudinal view of the carotid bifurcation (B‐mode image plus outline of the color area), the residual lumen diameter (*L*) and the presumed normal diameter (*A*) at the point of maximum stenosis were obtained and recorded. The percentage of stenosis was calculated with the following formula: (*A *− *L*) × 100/*A*.

The Doppler investigation was considered abnormal when increased peak systolic velocities (≥130 cm/sec for carotids or ≥40 cm/sec for vertebral arteries), or turbulent, demodulated, inverted, or absent flows were found at the carotid or vertebral level. In addition, a reduced diastolic component in the vertebral arteries (end diastolic velocity <30% of peak systolic velocity) was also considered abnormal.

### Statistical analysis

In univariate analysis, the continuous variables with normal distribution were described with mean ± standard deviation; otherwise, when distribution was not normal, median and interquartile range were used. The comparisons between means were assessed by Student's *t* test, while Mann–Whitney's nonparametric test was used to compare medians. The comparisons between percentages were tested by χ^2^.

The multivariate analysis of the factors predictive of dependency or death (mRS > 2) at 9 months was performed by multiple logistic regression, with backward elimination of the nonsignificant associations. Beta coefficients and their standard errors allowed the calculation of odds ratios and 95% confidence intervals.

The multivariate analysis of the factors predictive of mortality during the follow‐up period was performed by Cox proportional hazards regression, taking into account the time of observation (from Stroke Unit admission up to the date of death, or to the date of the phone interview to survivors). Also in this case, nonsignificant associations were removed by backward elimination procedure, and hazard ratios with 95% confidence intervals were calculated from the *β* coefficients and their standard errors.

Two‐tail tests were used throughout and *P* values < 0.05 were considered significant. To account for multiple testing in univariate analysis, *P* values were adjusted according to Bonferroni's method. Multiple‐testing adjusted *P* values (Pmta) are reported in the text, while standard *P* values are shown in the tables.

## Results

### First end‐point: modified Rankin scale ≥2

Table 1 shows patients' baseline characteristics, according to the possible subsequent outcome of dependency or death (mRS > 2). Thirty patients who had a mRS > 2 before the stroke were excluded from the analysis.

**Table 1:** Baseline characteristics of stroke patients according to mRs at follow‐up

	mRS ≤ 2 (*N* = 121)	mRS > 2 (*N* = 158)	*P* value
Age (years)	71.5 ± 10.6	79.1 ± 10.6	<0.0001
Male sex	75 (62.0)	71 (44.9)	0.005
TOAST classification (Adams et al. 1993)			
Large artery	13 (10.7)	23 (14.6)	0.35
Cardioembolism	16 (13.2)	52 (32.9)	0.0001
Small artery	53 (43.8)	23 (14.6)	<0.0001
Other determined cause	1 (0.8)	1 (0.6)	0.85
Undetermined cause	38 (31.4)	59 (37.3)	0.30
OCSP classification (Bamford et al. 1991)			
LACS	54 (44.6)	21 (13.3)	<0.0001
PACS	46 (38.0)	71 (44.9)	0.25
TACS	6 (5.0)	52 (32.9)	<0.0001
POCS	15 (12.4)	14 (8.9)	0.34
Initial NIHSS score	4 [2–7]	11 [6–17]	<0.0001
mRS before stroke	0 [0–0]	0 [0–0]	0.26
mRS at discharge	2 [1–4]	4 [4–5]	<0.0001
Hypertension	96 (79.3)	133 (84.2)	0.30
Diabetes	29 (24.0)	34 (21.5)	0.63
Hypercholesterolemia	72 (59.5)	75 (47.5)	0.05
Ex‐smoker	47 (38.8)	39 (24.7)	0.01
Current smoker	24 (19.8)	13 (8.2)	0.005
Ever smoker	71 (58.7)	52 (32.9)	<0.0001
Atrial fibrillation	16 (13.2)	59 (37.3)	<0.0001
Previous myocardial infarction	19 (15.7)	22 (13.9)	0.68
Previous stroke	13 (10.7)	24 (15.2)	0.28
Peripheral artery disease	7 (5.8)	10 (6.3)	0.85

LACS, lacunar anterior circulation syndrome; mRS, modified Rankin scale; NIHSS, National Institutes of Health Stroke Scale; OCSP, Oxfordshire Community Stroke Project; PACS, partial anterior circulation syndrome; POCS, posterior circulation syndrome; TACS, total anterior circulation syndrome; TOAST, Trial of ORG 10172 in Acute Stroke Treatment.

Values are mean ± SD, median [interquartile range], or number (percentage). The patients with mRS > 2 before the stroke (*N* = 30) were excluded from this analysis.

As expected, the patients with mRS > 2 at follow‐up were older than those with lower score (Pmta < 0.002). Also, among them there was a higher prevalence of women (Pmta = 0.12) (but at baseline, women were older than men: 78.1 ± 11.4 vs. 73.7 ± 10.7 years, *P* = 0.0007).

Among the patients with worse outcome, with respect to those with better outcome, there was a higher prevalence of cardioembolic strokes, atrial fibrillation, and TACS, while there was a lower prevalence of small cerebral artery impairment and lacunar syndromes (LACS). The higher NIHSS score in the patients with worse prognosis confirmed this expected tendency and, similarly, the mRS at discharge predicted the subsequent mRS at follow‐up (Pmta ≤ 0.002 for all these tests).

Of cardiovascular risk factors, hypertension and diabetes did not differ between the two groups, while there was a lower number of hypercholesterolemics (Pmta not significant), and especially of ever smokers (Pmta = 0.002), among the patients with worse outcome.

Of ultrasound parameters (Table 2; the patients with mRS > 2 before the stroke were also excluded from this analysis), the most significant difference concerned the prevalence of carotid stenoses ≥60%, which were more frequent in the patients with mRS > 2 (Pmta = 0.04). Overall, the patients with carotid stenosis ≥ 50% were 71. Of them, 40 had a stenosis ≥60% and, of the latter, 26 had a stenosis ≥70%. Moreover, we assessed separately the patients with stenosis ≥60% ipsilateral to the cerebral lesion (symptomatic, *N* = 20) and those with contralateral stenosis (asymptomatic, *N* = 20). Only the patients with ipsilateral stenosis had a significantly worse prognosis: 18 (11.4%) with follow‐up mRS > 2, versus two (1.7%) with mRS ≤ 2 (*P* = 0.002). Instead, the patients with contralateral stenosis did not have a worse prognosis: 13 (8.2%) with mRS > 2 versus seven (5.8%) with mRS ≤ 2 (*P* = 0.43).

**Table 2:** Carotid and vertebral ultrasound parameters according to mRS at follow‐up

	mRS ≤ 2 (*N* = 121)	mRS > 2 (*N* = 158)	*P* value
Carotid plaque	98 (81.0)	129 (81.6)	0.89
Carotid stenosis ≥50%	24 (19.8)	47 (29.7)	0.06
Carotid stenosis ≥60%	9 (7.4)	31 (19.6)	0.004
Carotid stenosis ≥70%	7 (5.8)	19 (12.0)	0.08
Irregular surface	6 (7.4)	13 (8.2)	0.28
Soft echostructure	11 (9.1)	7 (4.4)	0.12
Dense echostructure	57 (47.1)	73 (46.2)	0.88
Calcific echostructure	18 (14.9)	25 (15.8)	0.83
Mixed echostructure	29 (24.0)	55 (34.8)	0.05
Carotid Doppler abnormality^a^	10 (8.3)	29 (18.4)	0.02
Vertebral Doppler abnormality^a^	18 (14.9)	44 (27.8)	0.01

mRS, modified Rankin scale.

Values are number (percentage). The patients with mRS > 2 before the stroke (*N* = 30) were excluded from this analysis.

a Doppler abnormalities = increased peak systolic velocities, reduced vertebral diastolic component, or turbulent, demodulated, inverted, or absent flows.

Furthermore, the patients with worse prognosis had a higher prevalence of carotid plaques with mixed echostructure, and more frequent flow abnormalities both at the carotid and vertebral level (Pmta not significant for all).

To establish what associations with mRS > 2 at follow‐up were independent, a multiple logistic regression was performed including the following variables (*P* ≤ 0.05 in Tables 1 and 2): age, sex, LACS, TACS, initial NIHSS score, hypercholesterolemia, ever smoker, atrial fibrillation, carotid stenosis ≥60%, ipsilateral carotid stenosis ≥60%, mixed echostructure, abnormal Doppler carotid assessment, and abnormal Doppler vertebral assessment. To avoid collinearity and reduce the number of the covariates, in this analysis, cardioembolism, small artery, ex‐smoker and current smoker were not included, as they were strongly associated, respectively, with atrial fibrillation, LACS, and ever smoker. Also, mRS at discharge was not included due to its strict association with mRS at follow‐up. After backward elimination procedure, only the following baseline variables remained independently associated with the dependency or death end‐point: initial NIHSS score, age, and carotid stenosis ≥60% (Table 3). Ipsilateral stenosis ≥60% was eliminated, as well as the remaining variables. The relative risk (odds ratio) associated with carotid stenosis ≥60%, adjusted for NIHSS score and age, was 3.5 (95% C.I. 1.5–8.6).

**Table 3:** Baseline variables independently associated with a mRS > 2 at follow‐up

Variable	*β* coefficient	SE	χ^2^	*P* value
Initial NIHSS score	0.26265	0.03762	48.8	<0.0001
Age	0.06416	0.01524	17.7	<0.0001
Carotid stenosis ≥60%	1.26458	0.45558	7.7	0.006
Intercept	−6.72789	1.23420	29.7	<0.0001

LACS, lacunar anterior circulation syndrome; mRS, modified Rankin scale; NIHSS, National Institutes of Health Stroke Scale; TACS, total anterior circulation syndrome.

Result of a multiple logistic regression, with backward elimination procedure. The initial model included age, sex, LACS, TACS, initial NIHSS score, hypercholesterolemia, ever smoker, atrial fibrillation, stenosis ≥60%, ipsilateral stenosis ≥60%, mixed echostructure, carotid Doppler abnormality, vertebral Doppler abnormality. The patients with mRS > 2 before the stroke (*N* = 30) were excluded from this analysis. Overall, *R* ^2^: 0.31 – *P* < 0.0001.

### Second end‐point: mortality for any cause

Table 4 illustrates baseline patients' characteristics according to mortality at follow‐up. Sixty‐nine patients had died at the time of follow‐up, including 10 patients who had died during hospitalization, 34 further patients who had died within 90 days, and 25 patients who had died after 90 days. As expected, also this end‐point was strongly associated with age (Pmta < 0.002). Instead, there was no relationship with female sex, differently from what was seen for dependency. There was on the contrary a slightly nonsignificant higher prevalence of men among the deceased, despite their younger age compared to women.

**Table 4:** Baseline characteristics of stroke patients according to mortality at follow‐up

	Survivors (*N* = 240)	Deceased (*N* = 69)	*P* value
Age (years)	74.7 ± 10.9	81.9 ± 10.4	<0.0001
Male sex	123 (51.3)	37 (53.6)	0.73
TOAST classification (Adams et al. 1993)			
Large artery	32 (13.3)	8 (11.6)	0.70
Cardioembolism	45 (18.8)	32 (46.4)	<0.0001
Small artery	79 (31.7)	4 (5.8)	<0.0001
Other determined cause	2 (0.8)	0 (0)	0.45
Undetermined cause	82 (34.2)	25 (36.2)	0.75
OCSP classification (Bamford et al. 1991)			
LACS	77 (32.1)	3 (4.3)	<0.0001
PACS	110 (45.8)	15 (21.7)	0.0003
TACS	27 (11.3)	45 (65.2)	<0.0001
POCS	26 (10.8)	6 (8.7)	0.61
Initial NIHSS score	6 [3–10]	17 [12–22]	<0.0001
mRS before stroke	0 [0–0]	0 [0–1]	0.15
mRS at discharge	4 [2–4]	5 [4–5]	<0.0001
Hypertension	200 (83.3)	53 (78.8)	0.22
Diabetes	58 (24.2)	13 (18.8)	0.35
Hypercholesterolemia	130 (54.2)	24 (34.8)	0.005
Ex‐smoker	73 (30.4)	19 (27.5)	0.64
Current smoker	36 (15.0)	3 (4.3)	0.02
Ever smoker	109 (45.4)	22 (31.9)	0.05
Atrial fibrillation	51 (21.3)	37 (53.6)	<0.0001
Previous myocardial infarction	32 (13.3)	11 (15.9)	0.58
Previous stroke	36 (15.0)	9 (13.0)	0.68
Peripheral artery disease	15 (6.3)	2 (2.9)	0.28

LACS, lacunar anterior circulation syndrome; NIHSS, National Institutes of Health Stroke Scale; OCSP, Oxfordshire Community Stroke Project; PACS, partial anterior circulation syndrome; POCS, posterior circulation syndrome; TACS, total anterior circulation syndrome; TOAST, Trial of ORG 10172 in Acute Stroke Treatment; mRS, modified Rankin scale.

Values are mean ± SD, median [interquartile range], or number (percentage).

Mortality was associated directly with cardioembolism, atrial fibrillation, TACS, initial NIHSS score, and mRS at discharge, and inversely with small cerebral artery impairment, LACS, and partial anterior circulation syndromes (PACS) (Pmta ≤ 0.007 for all). In addition, there was a weak inverse relationship with current and ever smoking, and a stronger relationship, again of inverse type, with hypercholesterolemia (Pmta not significant for all).

Of ultrasound parameters (Table 5), vertebral flow abnormalities were strongly associated with mortality (Pmta < 0.001). No other parameter was significantly associated with this end‐point.

**Table 5:** Carotid and vertebral ultrasound parameters according to mortality at follow‐up

	Survivors (*N* = 240)	Deceased (*N* = 69)	*P* value
Carotid plaque	200 (83.3)	52 (75.4)	0.13
Carotid stenosis ≥50%	60 (25.0)	22 (31.9)	0.25
Carotid stenosis ≥60%	32 (13.3)	13 (18.8)	0.25
Carotid stenosis ≥70%	19 (7.9)	8 (11.6)	0.34
Irregular surface	17 (7.1)	4 (5.8)	0.71
Soft echostructure	19 (7.9)	2 (2.9)	0.14
Dense echostructure	113 (47.1)	29 (42.0)	0.46
Calcific echostructure	34 (14.2)	16 (23.2)	0.07
Mixed echostructure	70 (29.2)	21 (30.4)	0.84
Carotid Doppler abnormality^a^	34 (14.2)	11 (15.9)	0.71
Vertebral Doppler abnormality^a^	44 (18.3)	29 (42.0)	<0.0001

Values are number (percentage).

a Doppler abnormalities = increased peak systolic velocities, reduced vertebral diastolic component, or turbulent, demodulated, inverted, or absent flows.

To establish what variables were independently associated with mortality during the follow‐up period, a Cox proportional hazard regression was performed, which included the following variables (*P* ≤ 0.05 in Tables 4 and 5): age, sex, LACS, PACS, TACS, initial NIHSS score, hypercholesterolemia, current smoker, atrial fibrillation, and abnormal vertebral Doppler assessment. Sex was included, despite its nonsignificant *P* value in Table 4, because men's mortality was similar to that of women despite their younger age, which suggested an unfavorable role for male sex. Instead, cardioembolism, small artery, and ever smoker were not included as they were highly associated, respectively, with atrial fibrillation, LACS, and current smoker. Also, mRS at discharge was not included as it coincided with the end‐point (with the score 6) for the 10 patients who had died in stroke unit. The sample (*N* = 306) did not include three deceased patients whose date of death could not be obtained. After backward elimination procedure, only the following baseline variables remained independently associated with the death for any cause end‐point: initial NIHSS score, age, TACS, abnormal vertebral Doppler assessment, male sex, and hypercholesterolemia (the latter association was of inverse type) (Table 6). In particular, the relative risk (hazard ratio) of death associated with vertebral Doppler abnormalities, adjusted for the other five variables, was 2.2 (95% C.I. 1.3–3.6).

**Table 6:** Baseline variables independently associated with mortality for any cause at follow‐up

Variable	*β* coefficient	SE	χ^2^	*P* value
Initial NIHSS score	0.12364	0.02361	27.4	<0.0001
Age	0.04626	0.01570	8.7	0.003
TACS	0.94877	0.33101	8.2	0.004
Vertebral Doppler abnormality^a^	0.71428	0.25705	7.7	0.006
Male sex	0.64051	0.27344	5.5	0.02
Hypercholesterolemia	−0.55437	0.26530	4.4	0.04

LACS, lacunar anterior circulation syndrome; NIHSS, National Institutes of Health Stroke Scale; PACS, partial anterior circulation syndrome; TACS, total anterior circulation syndrome.

Result of Cox proportional hazard regression, with backward elimination procedure. Three deceased patients whose date of death could not be obtained were excluded from the sample. The initial model included age, sex, LACS, PACS, TACS, initial NIHSS score, hypercholesterolemia, current smoker, atrial fibrillation, vertebral Doppler abnormality. Overall, *R* ^2^: 0.37 – *P* < 0.0001.

a Vertebral Doppler abnormality = increased peak systolic velocity, reduced diastolic component, or turbulent, demodulated, inverted, or absent flow.

## Discussion

This study has shown that the carotid and vertebral ultrasound assessment, a routine investigation in acute stroke patients, not only has important short‐term implications in etiological stroke classification (Adams et al. 1993), in assessing the risk of recurrence (Rothwell 2008) and in suggesting possible revascularization procedures (North American Symptomatic Carotid Endarterectomy Trial Collaborators, 1991) but also has a relevant medium and long‐term prognostic significance, which is independent of the common prognostic indicators (NIHSS score and age). In particular, we demonstrated that a stenosis ≥60% in the main carotid axis is associated with an elevated risk of dependency or death, independent of cerebral lesion side, while flow abnormalities in the vertebrobasilar system are associated with a significant increase in mortality.

### Carotid stenosis ≥60%

In our sample, carotid stenoses ≥60% (as assessed by the ECST method) were more significantly associated with dependency or death than stenoses ≥50 and ≥70%. The lack of significance for the association between stenoses ≥70% (which were part of the stenoses ≥60%) and dependency or death was probably because of the low number of such stenoses. In univariate analysis, among stenoses ≥60%, only those ipsilateral to cerebral lesion were significantly related to prognosis. This was probably a consequence of the main relationship, that is, the one between cerebral impairment severity and prognosis. In fact, in multivariate analysis ipsilateral stenoses were eliminated, while all carotid stenoses ≥60% (ipsilateral and contralateral together) remained associated with prognosis. This relationship was strong (odds ratio 3.5) and independent of stroke severity and age.

This suggests that stenoses ≥60%, wherever located in the main carotid axis, are associated with a relevant impairment of cerebral circulation, or that they witness a general predisposition for atherosclerosis, with important prognostic implications in stroke patients.

### Vertebral Doppler abnormalities

Although ultrasound examination is not a validated method for vertebral artery imaging, the simple finding of vertebral Doppler abnormalities (i.e., increased flow velocities, reduced diastolic component, or turbulent, demodulated, inverted, or absent flows) was independently associated with the risk of death for any cause, with the highest level of significance after that of the indicators of stroke severity (NIHSS score and TACS) and of age. To the best of our knowledge, this is the first report of this association. Possibly, vertebral flow might be altered not only in case of vertebrobasilar stenosing lesions but also in the presence of obstacles to the arterial flow in the polygon of Willis, thus behaving as a signal of a wider impairment of intracranial circulation. A signal not to be undervalued as it is associated with doubling of death risk (hazard ratio 2.2).

### Other variables

In univariate analysis, female sex was significantly associated with the risk of dependency or death. This apparent relationship was because women were older than men and in fact in multivariate analysis, sex was eliminated from the variables independently associated with a mRS > 2, while age was retained. Vice versa, considering the mortality end‐point, male sex in univariate analysis was apparently associated with the same mortality as female sex, but men were younger than women. Therefore, the subsequent multivariate analysis attributed a higher death risk to male sex.

In the elderly, hypercholesterolemia has a paradoxical protective effect on mortality for any cause. This is a classical example of “reverse epidemiology”, already described in some previous studies (Kalantar‐Zadeh et al. 2004; Roquer et al. 2012), which probably reflects the prevalence, in the elderly, of the risk associated with malnutrition and frailty over the classical cardiovascular risk. In addition, there is also the possibility that statins, which are normally prescribed to hypercholesterolemic stroke patients, may have a favorable effect on prognosis (Amarenco et al. 2006).

As far as the apparent protective effect of smoking is concerned (the so‐called “smoke paradox” [Ali et al. 2013]), the hypothesis that not smoking may cause some disadvantage is obviously untenable. It seems probable, instead, that the patients who can smoke are those in better health conditions or, vice versa, that the most compromised and elderly patients avoid smoking. “Not smoking” might thus behave, in some cases, as an indicator of impairment of health status or of advanced age. In addition, smoking favors particularly lacunar strokes (Muscari et al. 2013), which are known to be associated with a better outcome compared to other types of stroke (as confirmed also by this study). It is probably for these reasons that in multivariate analysis smoking was eliminated and not confirmed as an independent “protective” factor.

### Limitations

The main limitation of this study is its retrospective design, which might have allowed some variations in the management of patients and their ultrasound assessment during the 22 months of data collection. To generalize our findings a prospective, possibly multicenter, study with standardized protocol will be necessary.

For the deceased patients, we could not record the cause of death, as this was not reliably provided by proxies or relatives during the telephone interview.

Finally, our patients were rather old, so our results might not be confirmed in younger stroke patients.

## Conclusions

This study has confirmed that the main prognostic indicators of stroke are age and the severity of neurological impairment (as reflected by NIHSS score) (Weimar et al. 2004; Muscari et al. 2011). However, some parameters of carotid and vertebral ultrasound assessment, which are easily obtainable in almost all ischemic stroke patients, may have a prognostic significance independent of these two indicators. In particular, carotid stenoses ≥60%, ipsi‐ or contralateral to cerebral lesions, were associated with an increased probability of dependency or death, and flow abnormalities in the vertebrobasilar system were a significant indicator of death risk. These signals should not be undervalued, because they may identify a subgroup of patients to follow with particular attention, whose outcome might improve with revascularization procedures in the cerebrovascular territory.

## Conflict of Interest

The authors have no conflict of interest to declare.
